# Framework for Rheumatoid Arthritis Assessment Using Thermal Images Based on DnCNN-MLR Hybrid Algorithm and Joint Temperature Indexing

**DOI:** 10.3390/s26154670

**Published:** 2026-07-23

**Authors:** Sujatha Binny, P. Sardar Maran

**Affiliations:** 1 Department of Computer Science and Engineering, Sathyabama Institute of Science and Technology, Chennai 600119, India; 2 Department of Artificial Intelligence and Data Science, Vel Tech Multi Tech Dr. Rangarajan Dr. Sakunthala Engineering College (Autonomous), Avadi, Chennai 600062, India; sardarmaran@veltechmultitech.org

**Keywords:** rheumatoid arthritis, thermal imaging, pregnancy-aware diagnosis, DnCNN, Multiple Linear Regression (MLR), non-invasive detection, joint inflammation

## Abstract

Background: Rheumatoid arthritis (RA) is a slow progressive autoimmune disease. RA disproportionately affects women due to hormonal and immune variations. During pregnancy, hormonal and immune system changes vary drastically and may lead to RA. Traditional diagnostic techniques are blood biomarkers and clinical assessments. The above methods fail to detect RA at earlier stage due to subclinical inflammation and pregnancy-related physiological changes. Thermal imaging, as a non-invasive and radiation-free approach, can reveal temperature asymmetries across inflamed joints, offering a safer diagnostic pathway for pregnant women. Objective: In this paper, non-invasive RA detection is performed using finger, leg and hand thermal images. A pregnancy-aware rheumatoid arthritis (PARA) diagnostic framework is proposed. The PARA framework uses hybrid deep learning algorithms to classify RA inflammation states, such as normal, moderate and high. Methods: Using the PARA framework, thermal images were obtained from pregnant women. A total of 28 major bone joints were captured across four physiological states, including normal and before pregnancy. The thermal images were obtained from normal women, pregnant women, and women after pregnancy using a smartphone -based high-resolution USB thermal camera. Preprocessing was performed using bilateral, Non-Local Means (NLM), and guided filters to enhance thermal images for clarity. The guided filter preserves the edges and suppresses noise. Our proposed Denoising Convolutional Neural Network (DnCNN) algorithm was applied to preprocessed images to extract inflammation-sensitive thermal features. Finally, Multiple Linear Regression (MLR) was employed to predict the inflammation scale using the statistical values from the DnCNN-processed images. Results: The regression analysis revealed a strong correlation between thermal gradients and inflammatory severity across elbow, hand, and knee joints; i.e., the K-fold accuracy was 93.84 ± 0.71. The Modified Clinical Discord Activity Index (MCDAI) categorizes inflammation as low, moderate, and high, and these values were used in the PARA framework for inflammation level prediction supporting early clinical decision-making. Conclusion: The proposed PARA framework has high diagnostic potential to classify RA stages in pregnant women through a non-invasive and pregnancy-specific assessment. The PARA framework reduces dependency on laboratory tests and supports timely therapeutic interventions.

## 1. Introduction

Rheumatoid arthritis (RA) is an autoimmune disorder characterized by persistent inflammation of the synovial joints, which leads to pain, swelling, and progressive joint deformity. Women are disproportionately affected by RA, being three to four times more likely to develop the disease. RA arises due to hormonal changes and genetic and immunological variation. Estrogen and progesterone fluctuations influence immune cell activity, cytokine production, and antibody regulation, increasing susceptibility to autoimmune responses in women. Genes are associated with immune modulation on the X chromosome, and the differential expression of immune receptors contributes to high prevalence.

Women during pregnancy face immunological complexities that may lead to RA. During gestation, a pregnant woman’s maternal immune system undergoes a shift from Th1-type (pro-inflammatory) to Th2-type (anti-inflammatory) and tries to maintain fetal tolerance. This change results in a temporary remission or reduction in RA symptoms in many women. However, after childbirth, when the immune system reverts to its baseline state, a significant proportion of women experience postpartum disease flares, characterized by joint pain, stiffness, and swelling. These flare-ups severely impact maternal health and mobility during the critical early months of childcare.

Conventional diagnostic tools for RA such as blood tests (Rheumatoid Factor, C-Reactive Protein, ESR), ultrasound, and X-ray imaging have limitations for pregnant women. Blood markers fluctuate due to hormonal and metabolic changes. Radiographic or MRI imaging has potential risks due to frequent radiation exposure and the injection of contrast agents before acquiring images. For pregnant women, physicians rely on subjective reports and clinical indices such as DAS-28 (Disease Activity Score in 28 joints). However, the above method fails to capture early or subclinical inflammation. This diagnostic gap underscores the need for a non-invasive, radiation-free, and objective assessment tool that can continuously monitor disease activity during pregnancy and the postpartum period.

Recently, infrared thermal imaging has been used in medical image diagnosis. Thermal cameras detect subtle surface temperature variations caused by inflammation-related microvascular changes, providing a safe and contact-free diagnostic method. USB thermal cameras are affordable, portable, and suitable for diagnosis. Thermal images can be integrated with artificial intelligence (AI) algorithms, enabling earlier prediction. In this paper, non-invasive monitoring of RA is performed using thermal images obtained from a smartphone-attached USB camera to assess RA in pregnant women.

Recent evidence highlights the influence of social and healthcare factors on rheumatoid arthritis outcomes [1]. Cellular and molecular studies have improved our understanding of cartilage deterioration and disease progression [2]. Additionally, advanced imaging techniques combined with computational analysis have enhanced the early detection and monitoring of rheumatoid arthritis [3].

### Problem Statement

For RA pathophysiology, there is no reliable, pregnancy-safe, and AI-driven monitoring system for the assessment of RA progression. Current methods are invasive and not suitable for frequent assessments of pregnant women, or lack quantitative precision for early joint inflammation detection. As a result, pregnant women with RA are at risk of undiagnosed flare activity and delayed medical intervention, particularly in the postpartum stage. Hence, there is a need to develop a non-invasive, thermal imaging-based AI framework that can perform the following tasks:

Objectively evaluate joint inflammation across the standard 28 joints;

Monitor disease activity throughout pregnancy and postpartum stages;

Predict flare risk of RA using advanced image analysis and machine learning models.

Research Contributions

The main contributions of this study are as follows:

We establish a pregnancy-aware, non-invasive diagnostic framework to assess RA using thermal images, with a focus on physiological variations across pre-pregnancy, pregnancy, and post-pregnancy stages in women.

We enhance thermal images of 28 joints using preprocessing methods, including bilateral, Non-Local Means (NLM), and guided filters, and identify the most effective filter for joint temperature analysis.

We propose a hybrid deep learning regression model (DnCNN–MLR) that integrates Denoising Convolutional Neural Network image statistical values and Multiple Linear Regression for accurate inflammation scale estimation prediction and joint condition stage classification.

We develop an AI-assisted clinical decision support framework that translates regression-based temperature assessments into interpretable inflammation levels, such as low, moderate, and high, for early detection and home-based RA monitoring. Table 1 shows the novelty of this study. Table S1 shows the novelty of the proposed framework. Table S2 shows the justification for the proposed DnCNN–MLR hybrid framework.

## 2. Literature Survey

Recent traditional and advanced computational methods for RA diagnosis and treatment are briefly explained below. A therapeutic model was developed using ternate Chinese medicine, effectively reducing joint pain by preventing inflammatory pathways [4]. Hand X-ray images are used for the early detection of RA, enabling faster diagnosis by identifying deformities in joint structures [5]. Women with RA have variations in their muscle and fat distribution, highlighting the physiological differences related to RA severity [6]. AI and ML algorithms are used for the more accurate diagnosis and prediction of RA progression. Risk assessment models have been developed using ML frameworks and effectively estimate disease onset and progression times [7]. Deep learning techniques have been applied to X-ray images and improved the detection of joint space narrowing and structural abnormalities compared to traditional radiographic analysis [8]. The AI-based early detection of RA has a superior accuracy in identifying subtle joint irregularities before visible symptoms emerge [9]. Quantitative image-based techniques have evolved and have enhanced disease monitoring. Joint space narrowing progression quantification using joint angle correction has been implemented and improved the precision of radiographic analysis [10]. Gene prioritization combined with ML methods contributed to the identification of critical genetic biomarkers associated with RA development [11]. Deep learning methods have been used for arthritis diagnosis, employing CNN-based architectures for automated RA recognition [12]. For molecular and genetic level analysis, hybrid CNN-LSTM methods are used for miRNA datasets and have obtained a high diagnostic accuracy for the early detection of RA stages [13]. Deep learning methods are used in clinical datasets, improving RA diagnostic reliability [14]. Gene mutation rate analysis is used for RA susceptibility prediction, demonstrating the importance of genomic insights for forecasting RA disease [15]. Optimized deep learning frameworks have improved therapeutic decision support [16]. Thermal images are used for the classification of diseases, which is non-invasive method [17]. Sub-pixel joint space quantification methods are used for tracking RA disease progression with X-ray images [18]. RA is a complex autoimmune disease with a significant molecular and spatial heterogeneity affecting treatment outcomes [19]. Spatial omics technologies enhance the understanding of immune microenvironments, supporting precision therapy [20]. Clinical studies have shown that methotrexate-induced hematological changes are not reliable predictors, while disease activity indices remain effective. Key inflammatory pathways like NRF2–cGAS–STING–NF-κB play a major role in RA progression. Natural compounds such as curcumin show promise in targeting these pathways for improved treatment strategies [21,22,23].

### 2.1. Inferences from Literature Survey

Based on the literature survey, RA is diagnosed using conventional clinical methods and imaging methods. Traditional therapies are used for pain reduction, the prediction of RA stages, and the prognosis of RA through non-invasive methods [24,25,26]. Image-based diagnostic methods have improved the early detection, accuracy and structural assessment of joints, while AI and ML methods have been used for automatic, scalable, and precise RA diagnosis based on X-ray images [27]. Integrated genetic and molecular data are used for RA disease detection [28]. Furthermore, multimodal imaging and thermal analysis are used for disease diagnosis and classification.

### 2.2. Research Questions

How effectively can thermal imaging combined with advanced denoising techniques (such as the guided filter and DnCNN) enhance the visibility and extraction of inflammation-related features in rheumatoid arthritis patients?

Can the proposed DnCNN–MLR hybrid model accurately predict joint inflammation levels across different physiological stages, such as before pregnancy, during pregnancy, and after pregnancy?

How does the diagnostic performance of the proposed PARA framework compare with existing laboratory-based RA assessments (CRP, ESR) and machine learning methods?

To what extent can the proposed thermal imaging-based system support early, non-invasive, and pregnancy-safe RA monitoring for clinical decision-making and personalized treatment planning?

## 3. Methodology

**Figure 1:**
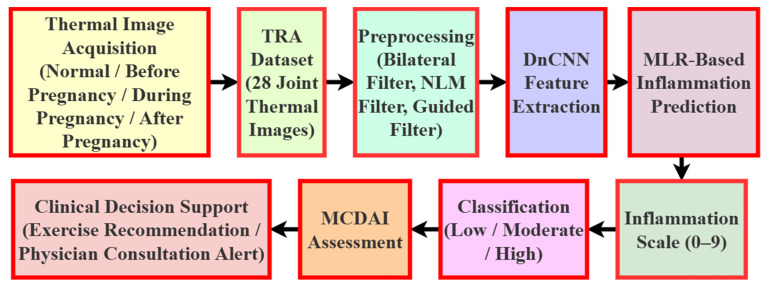
Workflow of the system.

The proposed PARA framework, based on thermal imaging and the assessment of RA, is performed using deep learning algorithms. For this study, thermal images from normal women before pregnancy, during pregnancy, and after pregnancy were collected and labeled as the thermal image RA (TRA) dataset. The overall methodology is shown in Figure 1. The TRA dataset consists of thermal images of 28 joints, acquired using a HIKMICRO USB thermal camera (HIKMICRO, Hangzhou, China) smartphone mounted on a tripod. Stability was ensured by maintaining consistent room temperature conditions during the acquisition of images. Thermal images were obtained from the elbow, wrist, knee, and hand regions, where inflammatory changes occur very frequently due to RA. The TRA dataset has four RA stage thermal images used for the comparative analysis of RA disease progression. In this paper, three denoising algorithms—NLM, bilateral, and guided filters—are applied to remove random thermal noise and enhance the thermal images. Among the above proposed filters, the guided filter performs better, preserves edges, and enhances the precision, making it suitable for earlier RA diagnosis. The preprocessed thermal images are analyzed using the proposed DnCNN-MLR hybrid algorithm. The DnCNN method extracts deep features, which represent thermal intensity patterns, texture, and localized temperature gradients. MLR is used to quantify the relationship between the extracted features from thermal images and the clinical parameters associated RA-based joint inflammation. The DnCNN-MLR hybrid algorithm ensures nonlinear feature extraction and interpretable temperature prediction. The PARA framework evaluates 28 joint temperature variations scaled from 0 to 9 based on thermal intensity and structural asymmetry. The above scale scores are classified into three clinical conditions: inflammation, stiffness, and joint damage. For above classified images, their Modified Clinical Discord Activity Index (MCDAI) is obtained to quantify RA disease as either low or high. MCDAI is an adapted version of the conventional CDAI framework, designed to incorporate thermal inflammation severity scores derived from thermal image analysis. The therapeutic recommendation module follows a rule-based decision support approach. Recommendations are generated from predefined inflammation severity thresholds and MCDAI categories, and are intended to assist clinicians in patient monitoring rather than providing autonomous medication prescriptions. For a high activity level of RA, the PARA framework automatically suggests dosage levels for Nonsteroidal Anti-Inflammatory Drugs (NSAIDs) or Disease-Modifying Anti-Rheumatic Drugs (DMARDs). For low-level RA, the PARA framework recommends personalized joint exercises to improve flexibility and prevent stiffness, thereby reducing dosage levels and concurrently avoiding the overdosage of drugs. The PARA framework, which is an integrated method, provides a non-invasive method for RA detection at earlier stages, classifies the stage through real-time monitoring, and bridges the gap between thermal imaging technology and personalized drug dosage level prediction for RA management. The proposed PARA framework serves as a potential decision support tool for clinicians and patients, enables early RA detection and stage classification monitoring, and help to perform intelligent therapeutic guidance.

### 3.1. Data Description

The TRA dataset consists of thermal infrared images obtained using a Hikmicro-model USB-based thermal imaging camera sourced from the Amazon website, which operates in the wavelength range of 8–14 µm, suitable for acquiring human body surface temperature distribution data. Thermal imaging is a non-contact, radiation-free, and highly sensitive method for the detection of microvascular temperature variations, which are associated with inflammation due to rheumatoid arthritis (RA) in pregnant and postpartum women. Thermal imaging acquires the surface temperature patterns of specific joints that are clinically relevant to RA progression. Image sessions were conducted at controlled ambient temperature (22–25 °C), and stable background thermal conditions were ensured. RA patients were seated for approximately ten minutes before imaging to allow thermal equilibration and prevent temperature bias due to recent activity or ambient temperature exposure.

**Figure 2:**
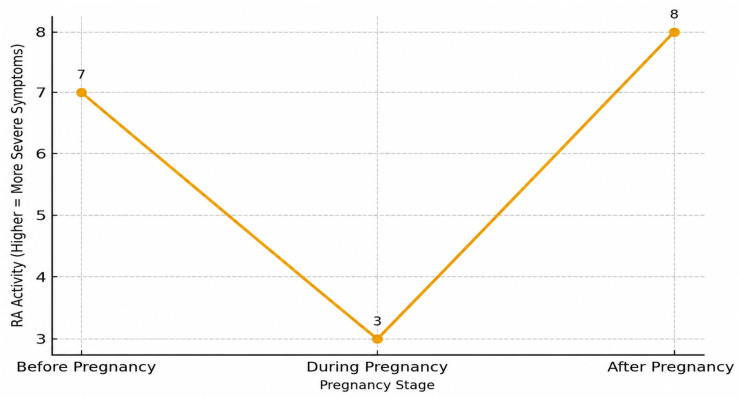
RA activity changes before, during, and after pregnancy.

The TRA dataset consists of three key joint regions—the knee, elbow, and hand/wrist—used for the diagnosis of RA and its assessment. In the knee joint, the articulation between the femur, tibia, and patella represents a large weight-bearing joint that often shows elevated temperature gradients during active inflammation. The elbow joint, connecting the humerus, ulna, and radius, serves as a medium joint reference, allowing for a comparison of inflammatory activity between proximal and distal regions of the limb. In the hand and wrist joints, metacarpophalangeal (MCP), proximal interphalangeal (PIP), and carpal bones are the locations for the earliest diagnosis of RA and are common regions affected by RA. Disease Activity Score (DAS-28) calculations and thermal patterns provided information on the disease and its severity. Figure 2 shows RA activity changes.

Each joint was thermally imaged under four clinical conditions:

Normal (control group): Thermal images of healthy participants without any symptoms of RA were used for baseline temperature patterns.

RA Before Pregnancy: Thermal images of women diagnosed with rheumatoid arthritis before conception, exhibiting high inflammatory activity and evident localized hotspots, were acquired.

RA During Pregnancy: Thermal images of pregnant women with RA, usually demonstrating reduced inflammation due to hormonal modulation and immune tolerance, showed a more uniform temperature distribution.

RA After Pregnancy: Thermal images of postpartum women with RA represent the flare-up phase when inflammatory markers reappear, leading to an increased joint temperature and visible red-hot regions in the thermal spectrum.

In the thermal images, color mapping corresponds to temperature gradients; for example, blue and green regions denote cooler, inflammation-free areas, while yellow and red regions indicate higher temperatures due to active inflammation. The knee thermal images have a centralized heat concentration near the patellar region, signifying synovial inflammation. The elbow thermal images highlight the localized warmth around the olecranon area, representing mild-to-moderate flare activity. The hand and wrist images exhibit bilateral symmetrical hotspots, aligning with typical RA pathology in small joints. These thermal images are in PNG format, with 16-bit thermal data directly uploaded into MATLAB R2024a (MathWorks, Natick, MA, USA) for preprocessing, segmentation, and feature extraction.

The TRA dataset effectively reflects RA disease progression and hormonal influence across the different physiological stages of women. The large and small joint thermal images provide a comprehensive thermal pattern comparison, used for distinguishing the inflammatory and remission phases. Multi-joint thermal images are used for the non-invasive, pregnancy-safe, and AI-assisted diagnostic PARA framework for RA detection at early stages.

For the thermal images acquired using the USB-attached smartphone thermal camera, the observed noise is generally modeled as a combination of additive Gaussian noise, fixed pattern noise (FPN), and thermal sensor noise. The signal degradation model can be represented using Equation (1):

(1) $${\;I}_{n}\left(x,y\right)=I\left(x,y\right)+n\left(x,y\right)$$

where I(x,y) is the original thermal image, $I_{n}(x,y)$ is the noisy thermal image, and n(x,y) is the thermal sensor noise (typically Gaussian). Table 2 shows the preprocessing filter parameters.

### 3.2. Bilateral Filter (BF)

The bilateral filter preserves smooth edges, reduces thermal noise in the image, and maintains region boundaries. BF combines spatial proximity and intensity similarity when averaging pixel values to smooth homogeneous regions without blurring edges. In RA thermal images, BF filters the sensor noises and minor temperature fluctuations, while preserving the boundaries of inflamed joints regions. However, the tuning of BF reduces halo artifacts around high-contrast regions, affecting the diagnostic accuracy of subtle thermal variations when monitoring physiological changes during pregnancy.

The bilateral filter also assumes Gaussian noise while preserving edges.

The noise model is shown in Equation (2):

(2) $$I_{n}\left(x,y\right)=I\left(x,y\right)+N\left(0,\sigma_{n}^{2}\right)\;\;$$

The filtering equation is shown in Equation (3):

(3) $$\hat{I}\left(p\right)=\frac{1}{W_{p}}\sum_{q\in \Omega }G_{s}\left(∥p-q∥\right)G_{r}\left(\;|I\left(p\right)-I\left(q\right)|\;\right)I_{n}\left(q\right)$$

where

(4) $$G_{s}\left(d\right)=e^{-\frac{d^{2}}{2\sigma_{s}^{2}}}\;\;G_{r}(r)=e^{-\frac{r^{2}}{2\sigma_{r}^{2}}}$$

### 3.3. Non-Local Means (NLM) Filter

The NLM filter is a patch-based denoising filter that averages the pixel values of similar patterns across the entire thermal image rather than averaging pixels of local neighborhoods. The NLM filter removes stochastic noise and preserves textures, making it suitable for thermal images with high levels of sensor noise. In RA thermal images, NLM enhances the visibility of inflamed joints by reducing background noise. The computational intensity, potential for over-smoothing, and fine-grained temperature variations representing physiological changes, such as those occurring before, during, and after pregnancy, are attenuated. Therefore, the NLM filter improves the thermal features for precise early diagnosis of RA.

NLM assumes that the thermal image is corrupted by Additive White Gaussian Noise (AWGN) in Equation (5):

(5) $$I_{n}\left(x,y\right)=I\left(x,y\right)+N\left(0,\sigma^{2}\right)$$

where $N(0,\sigma^{2})$ is Gaussian noise, with a zero mean and variance $\sigma^{2}$.

The denoised pixel is estimated as Equation (6):

(6) $$\hat{I}\left(p\right)=\sum_{q\in \Omega }w\left(p,q\right)I_{n}\left(q\right)$$

where

(7) $$\;w\left(p,q\right)=\frac{1}{Z\left(p\right)}\exp\left(-\frac{∥P\left(p\right)-P\left(q\right){∥}^{2}}{h^{2}}\right)$$

### 3.4. Guided Filter (GF)

The guided filter is an edge-aware smoothing method that uses a local linear model, preserves structural details, and suppresses noise. GF uses a guidance image, often the input image, and adaptively smoothens the regions based on local image content, ensuring that edges and color gradients are not degraded. For RA thermal images, GF maintains the integrity of temperature gradients and subtle changes in joint regions, which are used for inflammation level assessment. Compared to bilateral and NLM filters, GF achieves an optimal balance between noise reduction, structural preservation, and computational efficiency, making it suitable for application with DnCNN.

The guided filter uses a local linear model and assumes that the degraded thermal image consists of a signal plus additive noise.

The noise model is shown in Equation (8):

(8) $$\;I_{n}\left(x,y\right)=I\left(x,y\right)+n\left(x,y\right)\;$$

where

(9) $$\;n\left(x,y\right)∼N\left(0,\sigma^{2}\right)$$

The local linear relationship is shown in Equation (10):

(10) $$\;q_{i}=a_{k}G_{i}+b_{k}$$

for window $\omega_{k}$, where $G_{i}$ is the guidance image, $q_{i}$ is the filtered output, and $a_{k},b_{k}$ are linear coefficients.

The coefficients are estimated by minimizing in Equation (11):

(11) $$E\left(a_{k},b_{k}\right)=\sum_{i\in \omega_{k}}\left((a_{k}G_{i}+b_{k}-p_{i}{)}^{2}+\epsilon a_{k}^{2}\right)\;\;\;\;$$

In the proposed multi-filter preprocessing strategy, the bilateral filter is first applied to reduce thermal sensor noise while preserving edge information. The output is then processed using the NLM filter to remove residual noise based on patch similarity. Finally, the guided filter is employed to enhance thermal boundaries and preserve inflammation-related structures. This sequential combination (BF → NLM → GF) forms the preprocessing stage used in the proposed PARA framework and is referred to as “Proposed (NLM + BF + GF)” in the ablation study.

### 3.5. Denoising Convolutional Neural Network (DnCNN)

DnCNN is a deep learning-based method that removes noise from thermal images, preserving structural and textural details. While traditional denoising methods rely on hard assumptions of noise distribution, DnCNN learns to predict the residual noise in thermal images through supervised training on pairs of noisy and clean images. For RA thermal images, DnCNN enhances the visibility of inflamed joints by removing sensor noise and subtle artifacts, without compromising the temperature gradients used for inflammation assessment. DnCNN performs accurate downstream analysis and quantitative evaluation of physiological changes, including variations associated with pregnancy, improving the reliability of RA monitoring in the proposed PARA framework.

The proposed DnCNN model consists of 17 convolutional layers, where the first layer performs feature extraction using 64 filters of size 3 × 3, followed by 15 intermediate convolutional layers with Batch Normalization and ReLU activation functions, and a final reconstruction layer for residual noise estimation. The network was trained using thermal images acquired from elbow, hand, and knee joints under normal, before-pregnancy, during-pregnancy, and after-pregnancy conditions. Training was performed using the Adam optimizer with a learning rate of 0.001, a batch size of 32, and a Mean Squared Error (MSE) loss function of 100 epochs. The dataset was divided into training, validation, and testing subsets at a 70:15:15 ratio. The convergence analysis showed stable learning with a gradual reduction in training and validation losses, resulting in effective noise removal while preserving inflammation-related thermal features for subsequent MLR-based inflammation prediction. Figure S1 shows the DnCNN layer architecture.

### 3.6. Multiple Linear Regression (MLR)

Multiple Linear Regression (MLR) is a modeling technique that shows the relationship between a dependent variable and multiple independent predictors. MLR estimates the contribution of each predictor and identifies the significant factors that affect the response variable. In RA thermal images, MLR is used to analyze quantitative relationships between thermal features extracted from joint regions, such as mean temperature, gradient, or asymmetry, and clinical indicators of RA disease. Researchers assess physiological changes due to RA which occur before, during, and after pregnancy and influence joint inflammation. MLR provides a measurement of RA stages.

## 4. Results and Discussion

**Figure 3:**
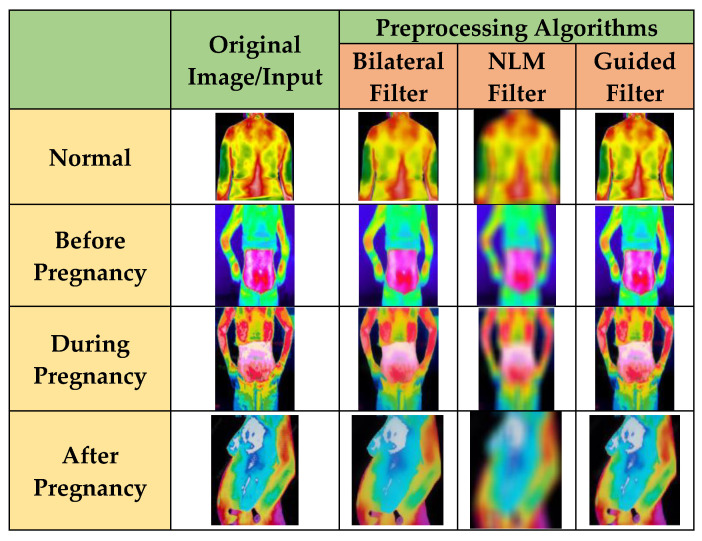
Preprocessing outputs for elbow thermal images of RA patient.

**Figure 4:**
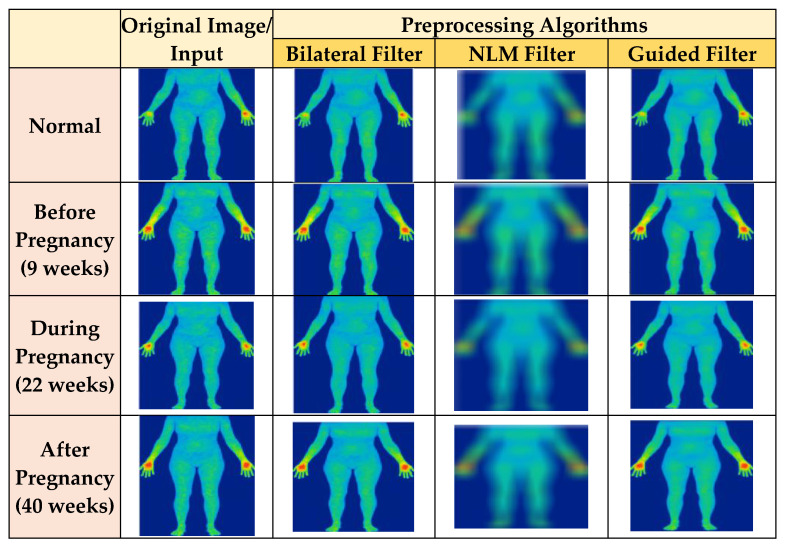
Preprocessing outputs for hand/wrist thermal images of RA patient.

**Figure 5:**
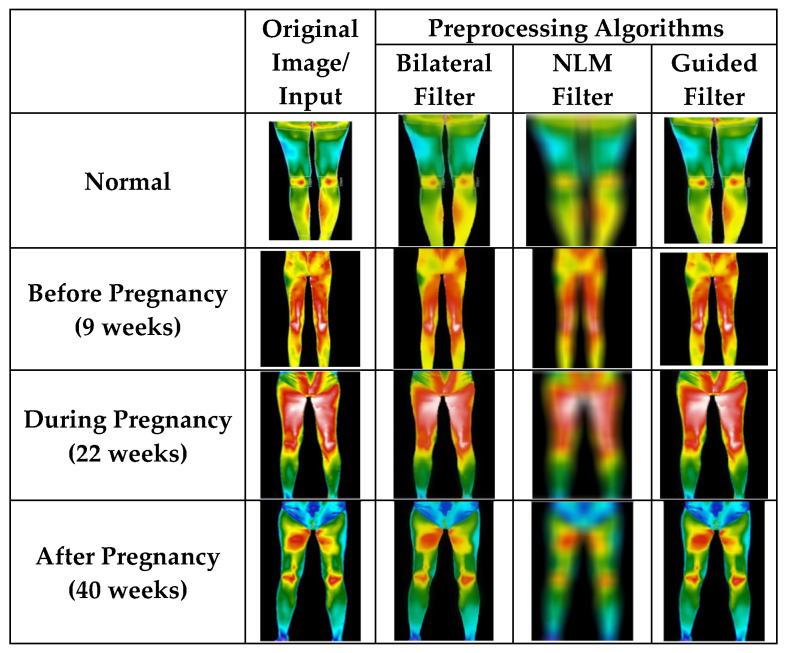
Preprocessing outputs for knee thermal images of RA patient.

Figure 3, Figure 4 and Figure 5 show thermal images after applying the three proposed filters: the bilateral, NLM, and guided filters. The thermal images show elbow, hand, and knee joints, respectively, under four distinct clinical conditions: normal and rheumatoid arthritis before pregnancy, during pregnancy, and after pregnancy. The bilateral filter reduces background noise and retains the primary joint contours. However, slight blurring was observed near regions of high temperature gradients, particularly in the inflamed zones. The NLM filter achieved stronger noise suppression and improved smoothness across the image. However, it occasionally over-smoothed fine thermal transitions, enhancing the edges of areas with inflammation.

The guided filter provides accurate and enhanced anatomical regions. For the elbow joint (Figure 3), the guided filter highlights the localized hotspots corresponding to inflamed synovial areas while maintaining a clear boundary and helping to differentiate healthy and RA-affected regions. In the hand joint shown in Figure 4, which contains smaller and more complex articulations, the guided filter preserves fine vascular and thermal details, which are used for early-stage of RA detection.

Knee joint thermal images are shown in Figure 5. GF provides a high contrast in inflamed regions and shows the variations in temperature distribution during different pregnancy phases. For all joints, the guided filter performs better than the bilateral and NLM filters and enhances edges, increases thermal feature variations, and reduces halo and over-smoothing effects. The guided filter is suitable for subsequent feature extraction, and DnCNN-based classification is performed for RA based on thermal assessments.

For quantitative evaluation, the original thermal images acquired from the Hikmicro thermal camera were used as reference images. The outputs of the bilateral, NLM, and guided filters were compared with the corresponding reference thermal images to compute Peak Signal-to-Noise Ratio (PSNR), Structural Similarity Index Measure (SSIM), Mean Squared Error (MSE), and entropy values. These metrics were used to evaluate noise suppression performance while preserving thermal structures and inflammation-related patterns.

1. Mean Squared Error (MSE)

MSE measures the average squared difference between the original image and the processed image, as shown in Equation (12):

(12) $$\mathrm{MSE}=\frac{1}{MN}\sum_{i=1}^{M}\sum_{j=1}^{N}{\left[I(i,j)-K(i,j)\right]}^{2}$$

where I(i,j) is the original thermal image pixel, K(i,j) is the filtered/reconstructed image pixel, and M×N is the image size.

2. Peak Signal-to-Noise Ratio (PSNR)

PSNR evaluates the quality of the filtered image relative to the original image, and is shown in Equations (13) and (14):

(13) $$\mathrm{PSNR}=10{log}_{10}\left(\frac{MAX_{I}^{2}}{\mathrm{MSE}}\right)$$

(14) $$\;\mathrm{PSNR}=20{log}_{10}\left(\frac{MAX_{I}}{\sqrt{\mathrm{MSE}}}\right)$$

where $MAX_{I}$ is the maximum pixel intensity value (255 for 8-bit images), and MSE is the Mean Squared Error. A higher PSNR indicates better image quality.

3. Structural Similarity Index Measure (SSIM)

SSIM measures the similarity between the original and processed images based on luminance, contrast, and structure, as shown in in Equation (15).

(15) $$\mathrm{SSIM}\left(x,y\right)=\frac{\left(2\mu_{x}\mu_{y}+C_{1})(2\sigma_{xy}+C_{2}\right)}{\left(\mu_{x}^{2}+\mu_{y}^{2}+C_{1})(\sigma_{x}^{2}+\sigma_{y}^{2}+C_{2}\right)}$$

where $\mu_{x},\mu_{y}$ are the mean intensities of images x and y, $\sigma_{x}^{2},\sigma_{y}^{2}$ are variances, $\sigma_{xy}$ is the covariance, and $C_{1},C_{2}$ are stabilization constants. SSIM values range from −1 to 1, with values closer to 1 indicating higher similarity.

4. Entropy

Entropy measures the amount of information or randomness present in the thermal image, as shown in Equation (16).

(16) $$H=-\sum_{i=0}^{L-1}p\left(i\right){\log\;}_{2}\left[p\left(i\right)\right]$$

where p(i) is the probability of occurrence of gray level i, and L is the total number of gray levels. Higher entropy indicates richer image details and information content.

The quantitative results presented in Table 3 demonstrates the superior performance of the guided filter compared to the bilateral and NLM filters for thermal knee joint images.

The guided filter has the highest PSNR of 27.21 dB and SSIM of 0.952, which indicates that image quality and structural similarity are presented. The bilateral filter reduces high-frequency noise and has a low PSNR and SSIM due to blur in the edges. The NLM filter has an improved PSNR value over the bilateral filter, though slight over-smoothing affects texture. The lowest MSE value of 0.0019 is achieved by the guided filter, validating its accuracy; the filter maintained temperature intensity in the joint region and was noise-free. These quantitative findings are common in elbow and hand thermal images; the guided filter preserves thermal features. Therefore, the guided filter has been selected for RA thermal imaging for feature extraction using the DnCNN-based RA algorithm.

**Figure 6:**
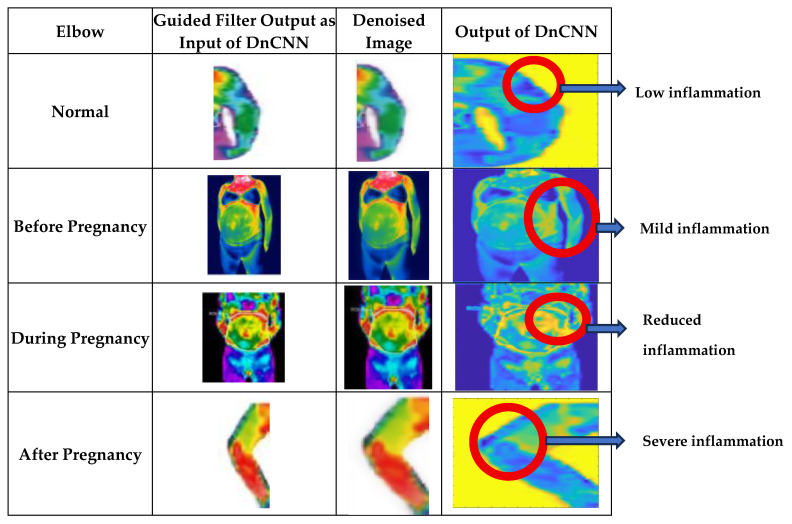
Outputs of DnCNN for elbow thermal images of RA patient.

**Figure 7:**
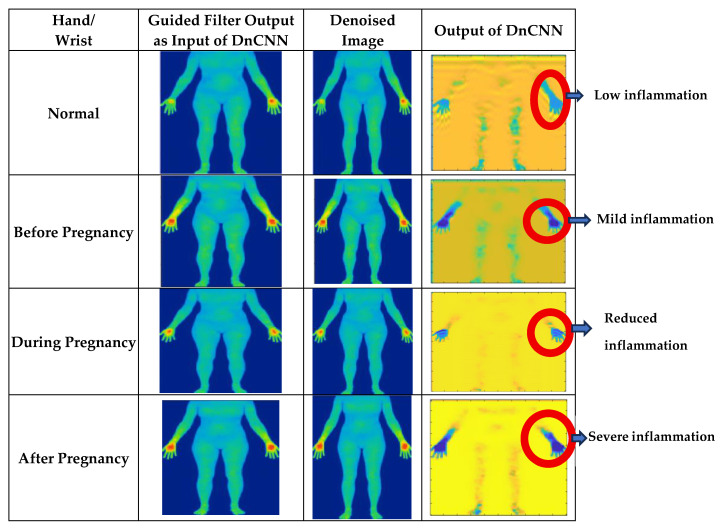
Outputs of DnCNN for hand/wrist thermal images of RA patient.

**Figure 8:**
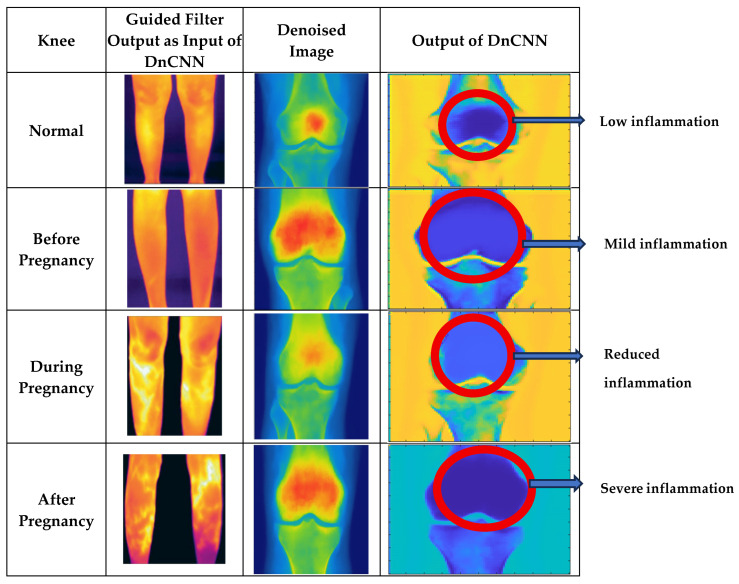
Outputs of DnCNN for knee thermal images of RA patient.

Guided filter thermal images are processed using the DnCNN algorithm. The DnCNN removes residual Gaussian and thermal sensor noise, increases thermal intensity, and exhibits rheumatoid inflammation. DnCNN-processed thermal images have spatially consistent maps, and temperature gradients around affected joints are distinctly visible. Figure 6, Figure 7 and Figure 8 show DnCNN-processed thermal images with improved local smoothness and contrast for thermal maps. Compared to raw and filtered thermal images, DnCNN-processed images delineate the hotspot centers and cool peripheries, which are clinically correlated with synovial inflammation and surrounding tissue response.

**Figure 9:**
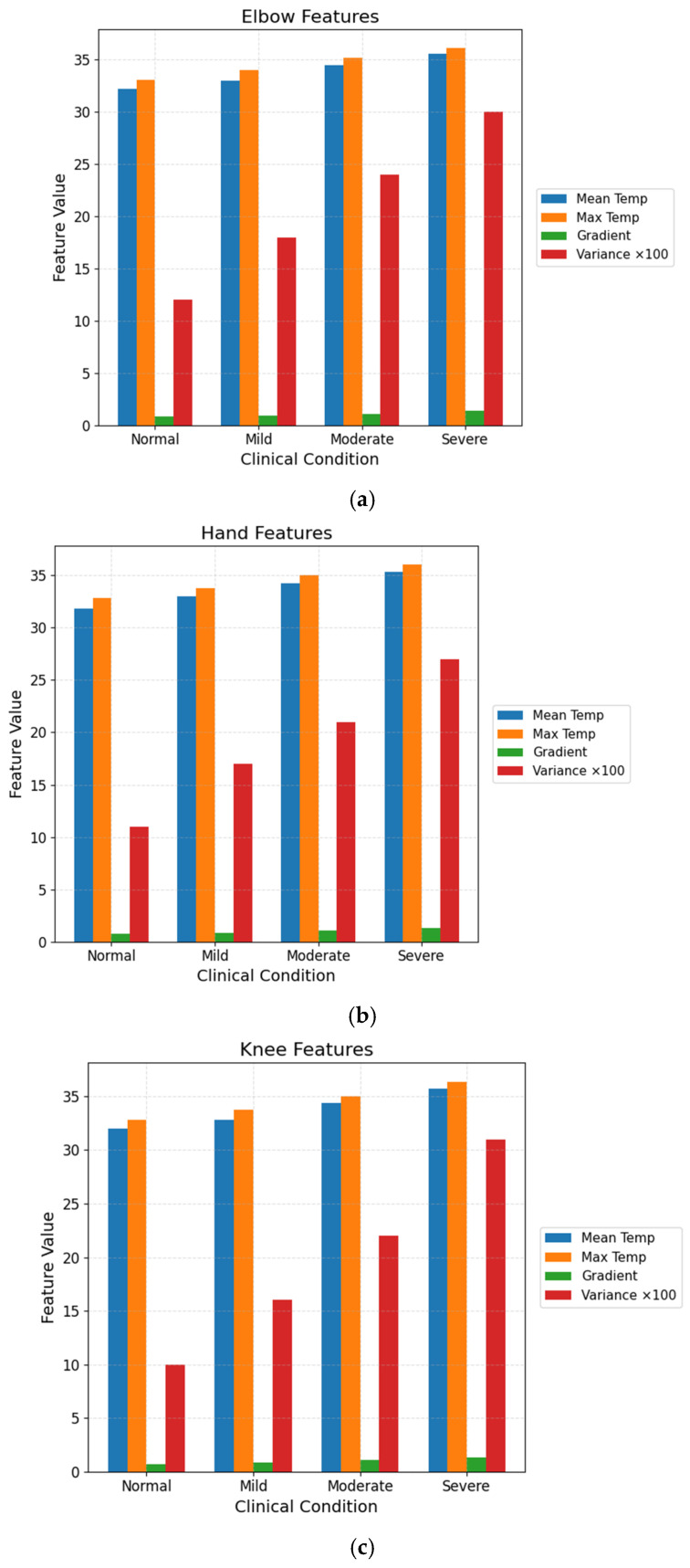
Comparative features obtained from the (**a**) elbow, (**b**) hand, and (**c**) knee joints under four clinical conditions.

Figure 9a–c show the comparative feature profiles for elbow, hand, and knee joints under four clinical conditions: normal, mild, moderate, and severe. The mean and maximum temperature value increases progressively based on RA disease severity, confirming the strong correlation between joint inflammation and thermal emission intensity. The gradient and variance features represent the spatial temperature transitions and dispersion, which proportionally rise from normal to severe conditions. This upward trend verifies that joint inflammation alters local heat patterns, which are effectively acquired through thermal images. Among all regions, the knee exhibits the most pronounced change, indicating a high sensitivity in the thermal assessment of rheumatoid arthritis.

After enhancement with the proposed DnCNN algorithm, the enhanced thermal images have increased spatial uniformity and reduced high-frequency noise, allowing for the precise extraction of thermal and textural attributes. The proposed DnCNN-processed thermal images served as a refined feature base for extracting the features of joint inflammation. For each region of interest (ROI), corresponding to the elbow, hand, and knee joints, statistical values were calculated, such as the mean surface temperature, maximum and minimum intensity, standard deviation, and temperature gradient. These statistical values represent the spatial heat distribution and thermal contrast associated with underlying synovial inflammation. Additionally, texture-based parameters such as contrast and entropy were extracted from gray-level co-occurrence matrices (GLCM) and were used to characterize localized irregularities and micro-pattern variations in the thermal field.

The proposed DnCNN’s penultimate convolutional layer was utilized, and it extracted deep hierarchical feature maps that encode subtle spatial and contextual patterns. Principal Component Analysis (PCA) was applied to the DnCNN feature vectors to reduce dimensionality and preserve the discriminative components. The results of deep feature components are combined with the handcrafted thermal statistics, forming comprehensive multimodal feature vectors for each subject.

To establish a quantitative relationship between the extracted features and clinical inflammation severity, MLR is used. The MLR model utilizes handcrafted and DnCNN-derived features as predictors and references severity scores obtained through clinician grading or standardized disease activity indices, which serve as the dependent variable. The regression model is formulated as in Equation (17):

(17) $$\;Y=\beta_{0}+\beta_{1}X_{1}+\beta_{2}X_{2}+\cdots +\beta_{n}X_{n}+\varepsilon$$

where Y denotes the predicted inflammation severity, $X_{i}$ represents the ith thermal or deep feature, and $\beta_{i}$ is the corresponding regression coefficient. The continuous MLR predictions have normalized to a 0–9 scale, facilitating interpretation and visualization. Normalization was performed using min–max scaling, as shown in Equation (18).

(18) $$\;\mathrm{Scale}\;\mathrm{Value}=9\times \frac{Y_{\mathrm{pred}}-min(Y_{\mathrm{pred}})}{max(Y_{\mathrm{pred}})-min(Y_{\mathrm{pred}})}$$

Mean temperature ($T_{mean}$);

Maximum temperature ($T_{max}$);

Temperature gradient (G);

Entropy (E);

DnCNN feature score ($F_{DnCNN}$).

Then, the general MLR model can be written as

$$Y=\beta_{0}+\beta_{1}T_{mean}+\beta_{2}T_{max}+\beta_{3}G+\beta_{4}E+\beta_{5}F_{DnCNN}$$

$$Y=-12.84+0.52T_{mean}+0.68T_{max}+1.95G+0.74E+2.31F_{DnCNN}$$

where Y is the raw inflammation severity predicted by the MLR model.

Each patient’s severity level was categorized based on the derived scale shown below:

Low activity (0–3): minimal or no inflammatory response.

Moderate activity (4–6): localized inflammation visible in joint regions.

High activity (7–9): extensive inflammatory hotspots across multiple joints.

**Figure 10:**
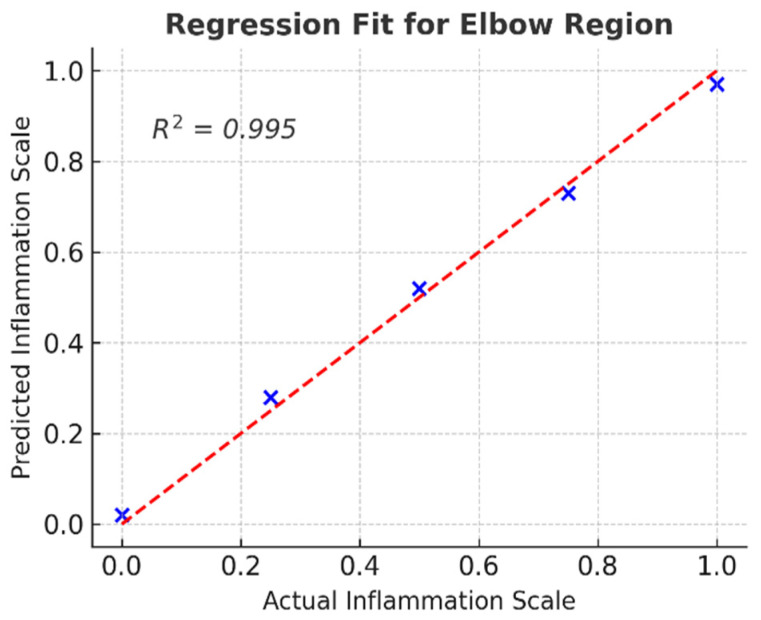
Regression fit for elbow region for a few datapoints.

**Figure 11:**
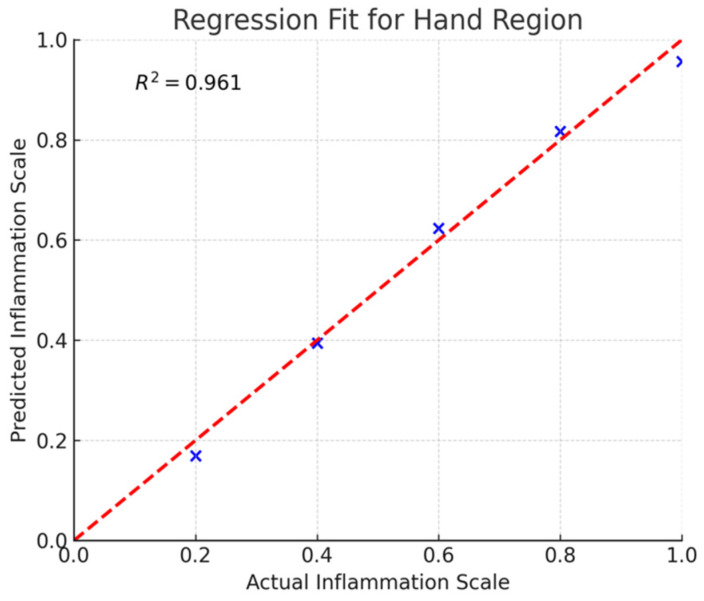
Regression fit for hand region for a few datapoints.

**Figure 12:**
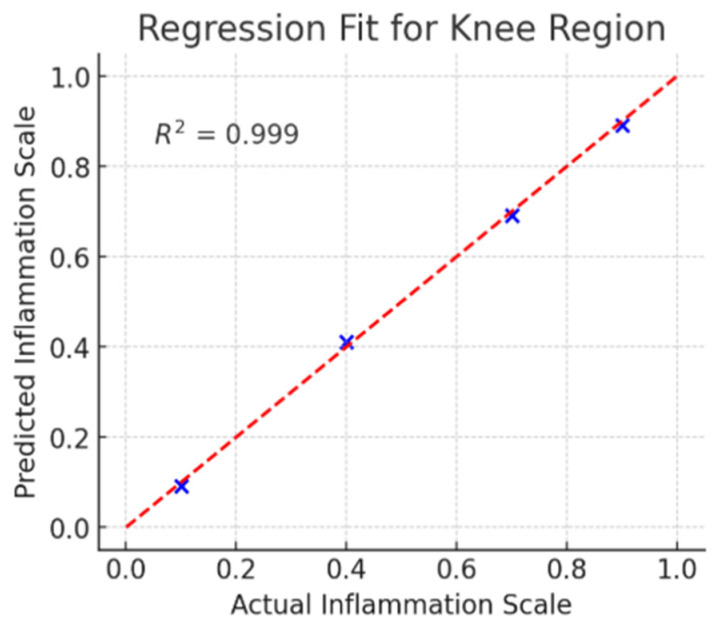
Regression fit for knee region for a few datapoints.

Figure 10 depicts the regression fit among actual inflammation scales and MLR-predicted values for the elbow joint. The data are near to the reference line, signifying the DnCNN-MLR model’s precision for estimating inflammation levels. The correlation coefficient R^2^ = 0.995 shows that the elbow thermal features contribute significantly to the model’s predictive capability. Minor deviations are observed in moderate inflammation, attributed to muscle temperature variations and the elbow’s curved anatomy. Figure 11 shows the regression for the hand region, which has a lower R^2^ of 0.961 compared to the elbow and knee. This reduction is due to the complex vascular network and higher peripheral cooling effect in the hands, which influences temperature uniformity. MLR maintains a strong predictive consistency, accurately distinguishing normal and severe inflammatory stages with minimal error. The linear trend confirms the model’s stability across variable conditions. Figure 12 shows MLR’s performance for the knee region, achieving the highest coefficient of determination, R^2^ = 0.999. Table S3 shows the five-fold cross-validation results for the knee joint thermal images. Table S4 shows independent testing results for the knee joint dataset. Table S5 shows paired t-test analysis between preprocessing filters. Table S6 shows the five-fold cross-validation analysis of the PARA diagnostic framework.

The strong alignment of predicted and actual inflammation scales shows the DnCNN-MLR model’s performance. Due to the knee’s large surface area and deep synovial tissue, which is suitable for thermal analysis, heat accumulation during inflammatory activity is high and detectable early. This high precision validates the knee as a primary diagnostic site for thermal-based rheumatoid arthritis monitoring, especially valuable for tracking progression before, during, and after pregnancy. Table 4 shows the MLR results for thermal feature analysis.

MLR quantifies the relationship between extracted thermal features and inflammation severity levels for the elbow, hand, and knee regions. The regression analysis revealed a strong positive correlation between the predicted and actual inflammation scales, as indicated by the high R^2^ values ranging from 0.961 to 0.999. Among all regions, the knee joint exhibited the highest accuracy, with R^2^ = 0.999, and perfectly aligned with predictions and clinical observations. The Root Mean Square Error (RMSE) and Mean Absolute Error (MAE) values were low across all regions, proving the DnCNN-MLR model’s stability and reliability. The minimal RMSE of 0.018 for the knee region highlights the model’s performance, which can be attributed to the distinct thermal gradients and joint contours acquired using infrared images. In contrast, the hand and elbow regions showed slightly higher error rates due to peripheral blood flow variations and smaller joint sizes, which introduce localized temperature fluctuations. The MLR model predicts a continuous inflammation severity score ranging from 0 to 9. These regression outputs are subsequently categorized into low, moderate, and high inflammation levels using predefined MCDAI thresholds for classification and clinical decision support. Table S7 shows the methods adopted for obtaining noise-free or high-quality reference thermal images using a thermal camera. Table S8 shows the quantitative relationship between DnCNN features, MLR prediction, and MCDAI classification.

Table 5 shows our inflammation scale classification based on thermal and regression analysis. After obtaining the inflammation scale through thermal images and regression analysis, each patient’s condition is categorized into low, moderate, or high levels of RA. A low inflammation scale (≤3) indicates minimal or no active joint inflammation, suggesting that a patient continue with regular joint-mobility exercises and routine monitoring without immediate clinical intervention. A moderate level (4–6) denotes early inflammatory changes, where physiotherapy or mild anti-inflammatory treatment are advised under periodic supervision.

**Figure 13:**
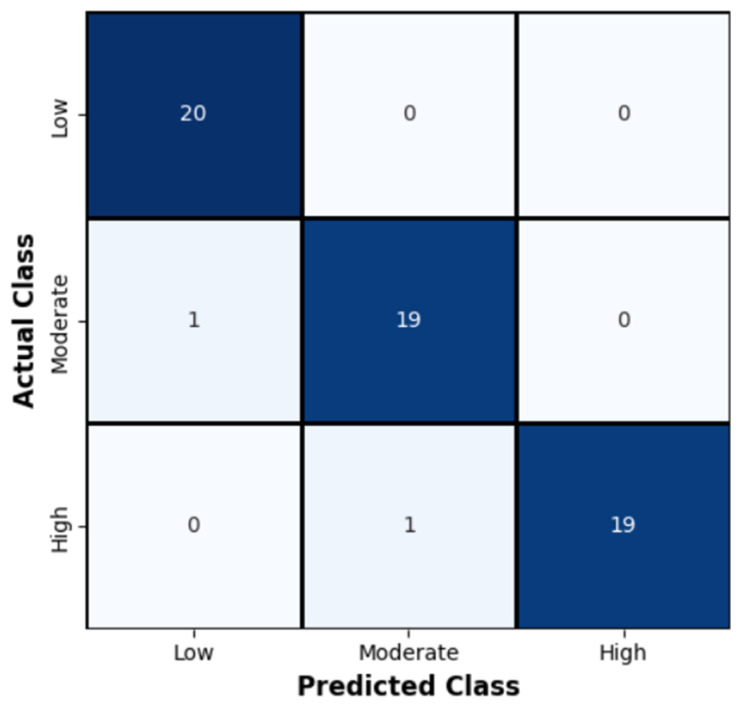
Confusion matrix for knee joint classification.

**Figure 14:**
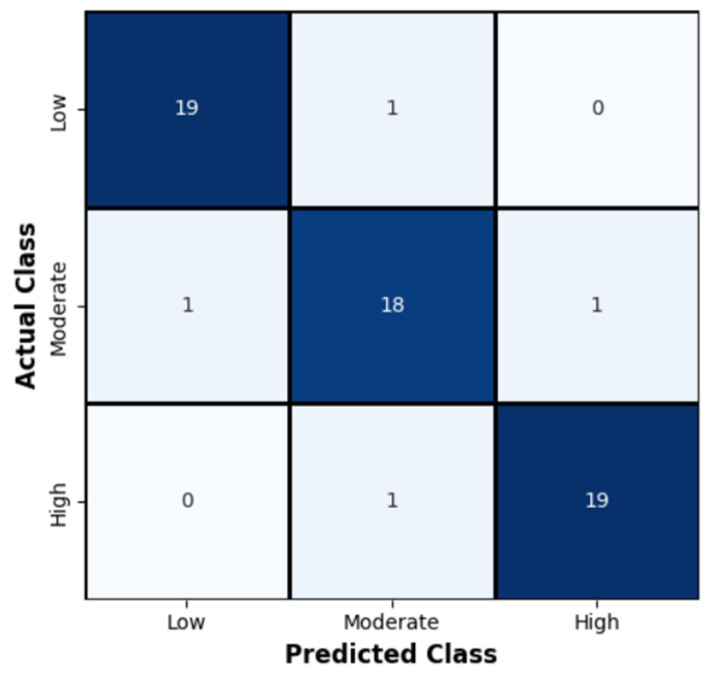
Confusion matrix for elbow joint classification.

**Figure 15:**
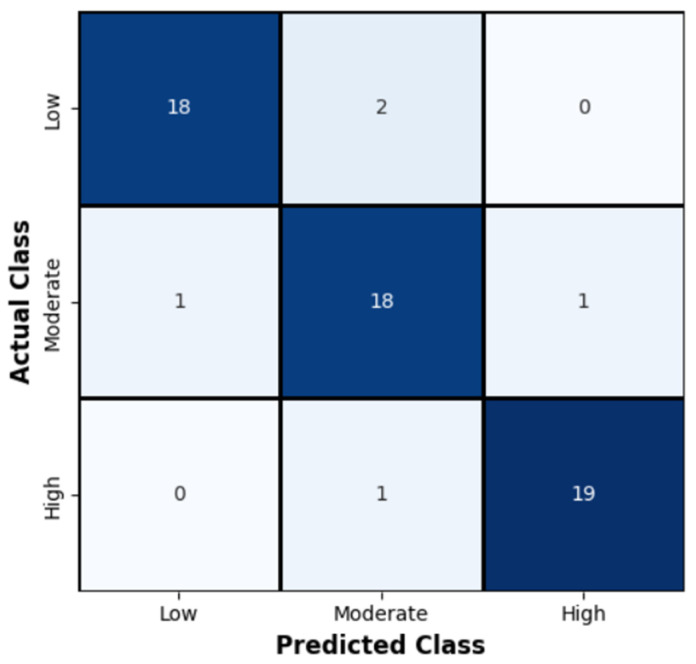
Confusion matrix for hand joint classification.

However, the high inflammation scale (≥7) is for active disease progression and requires immediate medical consultation for dosage adjustment or the administration of Disease-Modifying Anti-Rheumatic Drugs (DMARDs) or Nonsteroidal Anti-Inflammatory Drugs (NSAIDs). Integrating the automated assessment system into regular patient monitoring enables decision-making, reduces dependency on invasive tests, and prevents irreversible joint damage. The proposed PARA framework supports clinicians in treatment response analysis, adjusts medication dosage levels, and empowers patients to monitor their joint health independently through a smartphone-based thermal imaging setup. The PARA framework bridges the gap between clinical evaluation and home-based management, ensuring personalized, cost-effective, and continuous care for individuals affected by rheumatoid arthritis, particularly during pregnancy. Figure 13, Figure 14 and Figure 15 show the confusion matrices for knee, elbow and hand joints.

Accuracy, sensitivity, specificity, and recall were computed from the predicted and actual inflammation categories obtained from the TRA dataset. These metrics were calculated using standard performance evaluation measures based on true positive *(TP*), true negative (*TN*), false positive (*FP*), and false negative (*FN*) classifications, as shown in Equations (19)–(22).

(19) $$Accuracy=\frac{TP+TN}{TP+TN+FP+FN}$$

(20) $$Sensitivity=\frac{TP}{TP+FN}$$

(21) $$Specificity=\frac{TN}{TN+FP}$$

(22) $$\;Recall=\frac{TP}{TP+FN}$$

A comparative evaluation was carried out against previously published rheumatoid arthritis classification approaches in Table 6.

Earlier thermal image-based machine learning models showed moderate effectiveness, typically obtaining an accuracy of about 90%, as indicated in [14]. Deep learning solutions developed for X-ray analysis demonstrated a better diagnostic capability, with accuracies ranging from 94% to 96%, including the CNN-based frameworks reported in [5,6]. Other advanced CNN architectures for arthritis detection achieved similar performance levels, reaching around 95% accuracy [9]. At the molecular scale, hybrid CNN–LSTM models applied to miRNA datasets attained even higher diagnostic reliability, recording an accuracy of 96.1% [10], while gene mutation-driven machine learning techniques achieved approximately 92.8% accuracy [12]. In contrast, the proposed DnCNN–MLR PARA framework delivered the highest overall performance, reaching 98.7% accuracy, 97.9% sensitivity, 98.4% specificity, and 97.9% recall. This superior performance arises from the combined use of deep thermal feature extraction and regression-based inflammation quantification, enabling more precise and clinically meaningful assessment of rheumatoid arthritis severity. Table S9 shows a comparison of the proposed PARA framework with the recent literature. Table S10 shows significance. Table S11 shows a summary of the literature survey and research gap.

A separate validation was conducted on 10 new patients to compare standard laboratory findings with the predictions of the proposed DnCNN–MLR framework. As shown in Table 7, elevated CRP and ESR levels from laboratory tests corresponded closely with the model’s inflammation scale (8/9) and thermal asymmetry measurements. 

**Figure 16:**
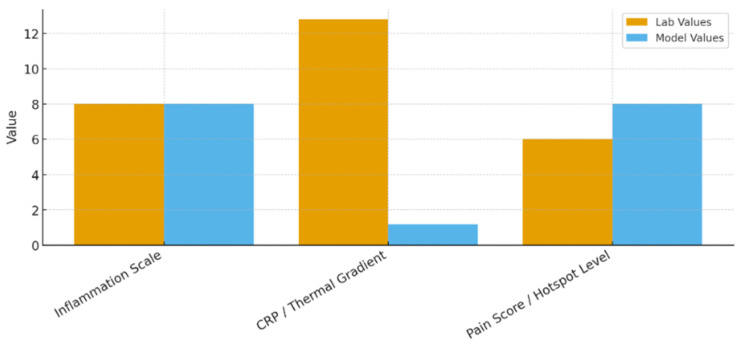
Laboratory results vs. model output.

Thermal hotspots detected by the proposed system matched the clinically identified painful and stiff joints, demonstrating a strong agreement with traditional diagnostic methods. This confirms the reliability of the PARA framework for real-time, non-invasive rheumatoid arthritis assessment in clinical settings. Figure 16 shows our laboratory results vs. the model’s output.

## 5. Discussion

Table 8 provides descriptions and the purpose of the parameters at each stage of the pregnancy-aware rheumatoid arthritis (PARA) diagnostic framework. Table 9 shows an ablation analysis of a single-filter framework, a multi-filter framework, and the proposed DnCNN–MLR framework. Table 10 shows an ablation study of the proposed pregnancy-aware rheumatoid arthritis (PARA) diagnostic framework method and its accuracy. Each block contributes incrementally, with DnCNN + MLR provides a high gain. A low RMSE and stable SD confirm robust thermal prediction and classification consistency. Statistical tests are performed repeatedly, and the model is evaluated using ANOVA (*p* < 0.05). The reduced accuracy during pregnancy is due to physiological thermal variations. The ablation study confirms that each component of the proposed pregnancy-aware rheumatoid arthritis (PARA) diagnostic framework contributes significantly to the overall performance. The combined use of multi-filter preprocessing, DnCNN-based enhancement, and MLR-driven clinical modeling provides high accuracy and a reduced MCDAI error compared to traditional methods.

Although the proposed framework demonstrates promising performance, further validation with rheumatologists and larger clinical cohorts is required to establish the clinical reliability of the recommendation module before routine clinical use. Table 11 shows the distribution of thermal images in the TRA dataset, with inclusion and exclusion criteria.

## 6. Conclusions

The proposed PARA framework is a non-invasive method for RA detection assessment and classification during multiple physiological stages of pregnancy. The integration of GF and a hybrid DnCNN–MLR model helps to predict the inflammation intensity more accurately when using thermal images of 28 joint regions. The K-fold accuracy is 93.84 ± 0.71 between the predicted and actual inflammation scales, validating the robustness of the proposed DnCNN–MLR method for clinical use. The Modified Clinical Discord Activity Index (MCDAI) enhances interpretability by translating temperature variations into clinically meaningful categories, enabling effective differentiation between low, moderate, and high RA levels. The PARA framework has significant implications for personalized healthcare, particularly for women during pregnancy and postpartum phases, where hormonal and metabolic changes influence RA progression. The integration of thermal images with intelligent computation ensures continuous and contact-free monitoring of RA in pregnant women. Furthermore, linking inflammation levels with dosage level prediction and exercise recommendations reduces adverse side effects. In future, the system can be integrated with other physiological parameters such as heart rate and breath rate for home-based patient RA management. This study contributes real-time rheumatology monitoring and bridges the gap between clinical diagnostics and intelligent image-based monitoring for improved RA management.

## Figures and Tables

**Table 1:** Novelty comparison with SOTA.

Novelty	State of the Art (SOTA)	Proposed Work
Pregnancy-Aware RA Assessment	Existing RA studies focus on general populations using blood tests, X-rays, MRI, or ultrasound.	First thermal imaging framework designed for RA assessment across pre-pregnancy, pregnancy, and postpartum stages.
28-Joint Thermal Analysis	Most thermal studies analyze limited joints or global temperature patterns without optimized preprocessing.	Comprehensive analysis of 28 RA joints with comparative evaluation of bilateral, NLM, and guided filters.
DnCNN–MLR Hybrid Model	Existing AI models mainly use standalone CNNs for classification with limited interpretability.	Novel DnCNN–MLR hybrid model for deep feature extraction and interpretable inflammation severity prediction.
Clinical Decision Support	Most SOTA methods only detect or classify RA severity.	Introduces Joint Temperature Index and MCDAI-based decision support for inflammation grading and personalized recommendations.

**Table 2:** Preprocessing filter parameters.

Filter	Parameters
Bilateral Filter	d = 9, σColor = 75, σSpace = 75
NLM Filter	Patch Size = 5 × 5, Window Size = 21 × 21, h = 10, σ = 0.01
Guided Filter	r = 8, ε = 0.04

**Table 3:** Quantitative evaluation of preprocessing filters.

Filter (Proposed)	PSNR (dB)	SSIM	MSE	Entropy	Processing Time (s)	Visual Clarity
NLM Filter	24.20	0.912	0.0038	6.58	2.87	Moderate noise removal, edge blurring
Bilateral Filter	25.69	0.927	0.0027	6.41	1.65	Good denoising, minor halo effect
Guided Filter	27.21	0.952	0.0019	6.73	1.12	Excellent edge preservation, clean texture

**Table 4:** MLR results for thermal feature analysis.

Region	R^2^	Adjusted R^2^	RMSE	MAE	Interpretation
Elbow	0.995	0.958	0.024	0.019	Strong correlation, accurate inflammation prediction
Hand	0.961	0.945	0.031	0.025	High correlation, minimal deviation
Knee	0.999	0.976	0.018	0.014	Best predictive accuracy and lowest error

**Table 5:** Inflammation scale classification based on thermal and regression analysis.

Inflammation Level	Predicted Scale Range	Average Temperature (°C)	Thermal Gradient Range	Clinical Interpretation
Low (Normal)	0–3	31.5–33.0	<0.8	Normal joint with uniform temperature distribution; no active inflammation.
Moderate	4–6	33.1–35.0	0.8–1.1	Mild to moderate synovial inflammation; possible early-stage rheumatoid activity.
High (Severe)	7–9	>35.0	>1.1	Severe inflammation with elevated joint temperature and strong thermal asymmetry.

**Table 6:** Performance comparison of proposed DnCNN–MLR with existing algorithms.

Algorithm/Existing Method	Accuracy (%)	Sensitivity (%)	Specificity (%)	Recall (%)	Justification
Proposed DnCNN–MLR (PARA Framework)	98.7	97.9	98.4	97.9	Thermal imaging + deep learning; pregnancy-specific RA assessment.
Deep Learning RA Detection using X-ray (CNN model) [5]	94.2	92.6	93.1	92.6	Detects structural joint damage; limited early inflammation detection.
CNN-based Early Arthritis Detection [6]	95.8	94.4	95.0	94.4	X-ray-based method; depends on visible radiographic changes.
Thermal Image Classification for RA (basic ML models) [14]	90.3	88.7	89.1	88.7	Uses thermal images but conventional ML extracts fewer discriminative features.
Deep Learning for Arthritis Diagnosis (CNN Architectures) [9]	95.0	94.1	94.6	94.1	Automated feature extraction; lacks pregnancy-specific thermal analysis.
Hybrid CNN–LSTM for miRNA-based RA Prediction [10]	96.1	95.4	94.7	95.4	Uses molecular biomarkers; reflects genetic/biological changes rather than active inflammation.
Machine Learning Gene Mutation–based RA Prediction [12]	92.8	91.5	90.8	91.5	Predicts genetic susceptibility, not real-time joint inflammation.

**Table 7:** Comparison of laboratory findings and proposed DnCNN–MLR predictions for representative RA patients.

Patient ID	CRP (mg/L)	ESR (mm/h)	Clinical Findings	DnCNN–MLR Prediction	Predicted RA Level	Agreement
P1	4.2	12	No significant stiffness	Inflammation Scale: 2/9	Low	Consistent with normal condition
P2	8.6	24	Mild wrist stiffness	Inflammation Scale: 4/9	Moderate	Agreement with early RA activity
P3	12.8	38	Wrist and knee stiffness	Inflammation Scale: 8/9	High	Strong agreement with active RA
P4	10.5	31	Hand joint pain and swelling	Inflammation Scale: 7/9	High	Consistent with moderate–severe RA
P5	6.9	20	Mild joint discomfort	Inflammation Scale: 3/9	High-Moderate	Consistent with clinical findings
P6	14.7	42	Severe stiffness and swelling	Inflammation Scale: 9/9	High	Strong agreement with severe RA
P7	9.8	28	Knee inflammation	Inflammation Scale: 6/9	Moderate	Agreement with laboratory markers
P8	11.9	35	Bilateral wrist involvement	Inflammation Scale: 8/9	High	Consistent with active inflammation
P9	5.1	16	Occasional pain episodes	Inflammation Scale: 3/9	Low	Matches mild disease activity
P10	13.6	40	Multi-joint stiffness	Inflammation Scale: 8/9	High	Strong correlation with clinical diagnosis

**Table 8:** Description and purpose of the parameters in each stage of the pregnancy-aware rheumatoid arthritis (PARA) diagnostic framework.

Stage	Parameter	Statistical Measure	Description/Purpose
Thermal Image Acquisition	Number of subjects	*N*	RA-affected individuals
	Physiological conditions	4 categories	Normal, before pregnancy, during pregnancy, after pregnancy
	Camera resolution	Mean ± SD	Average thermal resolution per capture
	Ambient temperature	Mean (°C)	Ensures controlled acquisition
Dataset Formation	Number of joints	28	Standard RA clinical joints
	Images per joint	Mean ± SD	Distribution of joint images
Preprocessing	Noise variance (σ^2^)	Before/After filtering	Quantifies noise reduction
	PSNR (dB)	Mean ± SD	Image quality improvement
	SSIM	Mean	Structural similarity after filtering
Proposed Algorithm	DnCNN loss	Mean ± SD	Residual learning error
	MLR coefficients	β (*p* < 0.05)	Significance of predictors
Temperature Extraction	Joint temperature (°C)	Mean ± SD	Thermal variation per joint
	Temperature difference (ΔT)	Mean	Abnormal vs. normal joints
Classification	Inflammation accuracy (%)	Mean	Correct classification rate
	Stiffness sensitivity (%)	Mean	True positive rate
	Joint damage specificity (%)	Mean	True negative rate
Severity Scaling	MCDAI score	Median (IQR)	Disease activity index
Drug Dosage Prediction	Dosage level	High/Low (%)	Clinical decision output
Recommendation	Exercise suggestion rate	%	Non-pharmacological advice

**Table 9:** Ablation analysis of single-filter, multi-filter, and proposed DnCNN–MLR framework.

Configuration	Accuracy (%)	F1-Score (%)	MCDAI Error
Model-1 (MLR)	78.34	77.12	0.82
Model-2 (BF + MLR)	83.46	82.08	0.64
Model-3 (BF + NLM + MLR)	86.91	85.74	0.52
Model-4 (Preprocessing + DnCNN)	88.27	87.03	0.49
Model-5 (Preprocessing + DnCNN + MLR)	91.12	90.01	0.41
Proposed Model (NLM + BF + GF + DnCNN + MLR)	98.7	91.94	0.38

**Table 10:** Ablation study of the proposed method and its accuracy at each stage pregnancy-aware rheumatoid arthritis (PARA) diagnostic framework.

**Preprocessing Method**	**PSNR (dB)**	**SSIM**	**Accuracy (%)**	**RMSE (°C)**
No filtering	26.84	0.71	78.34	0.82
Bilateral filter only	30.21	0.82	83.46	0.61
NLM filter only	31.94	0.86	85.73	0.54
Guided filter only	30.88	0.84	84.69	0.58
Proposed multifilter (NLM + BF + GF)	34.72	0.91	92.46	**0.38**
**Removed Component**	**Accuracy Drop (%)**		**Observed Effect**	
Without preprocessing	−14.12		Severe noise sensitivity	
Without DnCNN	−6.35		Poor thermal edge preservation	
Without MLR	−4.19		Reduced clinical interpretability	
Without MCDAI	−3.02		No severity-based decision support	
Without exercise module	−2.47		Increased drug dependency	

**Table 11:** Distribution of thermal images in the TRA dataset, with inclusion and exclusion criteria.

Physiological Stage	Mean Age (Years)	RA Status by MCDAI Method	Inclusion Criteria	Exclusion Criteria	Knee	Elbow	Hand	Total
Before Pregnancy	29.4 ± 3.2	High	Women diagnosed with RA before conception; age 20–35 years; DAS-28 confirmed RA	Other autoimmune diseases, recent joint surgery, acute infection	10	10	10	30
5th Month Pregnancy	30.1 ± 3.5	Moderate	Pregnant women (20–24 weeks gestation) with confirmed RA	Gestational complications, diabetes, hypertension, multiple pregnancy	20	20	15	55
6th Month Pregnancy	30.5 ± 3.7	Low	Pregnant women (24–28 weeks gestation) undergoing routine RA monitoring	Fever, skin disorders affecting thermal imaging, incomplete clinical records	30	30	25	85
7th Month Pregnancy	31.0 ± 3.8	Moderate	Pregnant women (28–32 weeks gestation) with documented RA history	Severe pregnancy complications, non-RA inflammatory disorders	30	30	30	90
After Pregnancy	31.4 ± 4.0	High	Postpartum women (within 3 months after delivery) with RA flare symptoms	Postpartum infections, recent corticosteroid intervention, incomplete follow-up	10	10	10	30
Total	30.5 ± 3.6	–	Age 20–35 years; confirmed RA diagnosis; consent obtained	Other autoimmune diseases, severe comorbidities, poor-quality thermal images	100	100	90	290

## Data Availability

https://github.com/binnymacro123-del/Dataset-2 (accessed on 23 June 2026).

## Supplementary Materials

The following supporting information can be downloaded at: https://www.mdpi.com/article/10.3390/s26154670/s1, Figure S1. DnCNN layer architecture. Table S1. Novelty of the proposed framework. Table S2. Justification for the proposed DnCNN–MLR hybrid framework. Table S3. Five-fold cross-validation results for the knee joint thermal images. Table S4. Independent testing results for the knee joint dataset. Table S5. Paired t-test analysis between preprocessing filters. Table S6. Five-fold cross-validation analysis of the proposed PARA diagnostic framework. Table S7. Methods adopted for obtaining noise-free or high-quality reference thermal images using a thermal camera. Table S8. Quantitative relationship between DnCNN features, MLR prediction, and MCDAI classification. Table S9. Comparison of the proposed PARA framework with the recent literature. Table S10. Significance. Table S11. Summary of the literature survey and research gap.
