# Supplementary material for: Framework for Rheumatoid Arthritis Assessment Using Thermal Images Based on DnCNN-MLR Hybrid Algorithm and Joint Temperature Indexing
Source: Sensors (Basel). 2026 Jul 23;26(15):4670. doi: 10.3390/s26154670 (PMC13468700; doi:10.3390/s26154670)
Supplement: Supplementary file 1 [file sensors-26-04670-s001.zip › sensors-4394764-supplementary.pdf]

Supplementary material

Table S1. Novelty of the proposed framework

S. No.	Novel Contribution
1	Development of a pregnancy-aware thermal imaging framework for non-invasive RA assessment.
2	Comprehensive 28-joint thermal analysis with optimized Guided Filter-based enhancement.
3	Integration of DnCNN and MLR for accurate and interpretable inflammation severity prediction.
4	AI-assisted clinical decision support using Joint Temperature Index and MCDA-based inflammation grading.

Table S2. Justification for the proposed DnCNN–MLR hybrid framework

Method	Strengths	Limitations	Advantage of Proposed DnCNN–MLR Framework
CNN	Automated feature learning	Limited interpretability	Provides interpretable inflammation prediction through regression coefficients
ResNet	Strong feature extraction capability	Requires larger datasets and higher computational cost	More suitable for limited thermal imaging datasets
EfficientNet	Efficient deep feature learning	Black-box prediction mechanism	Produces clinically interpretable inflammation scores
Random Forest	Robust nonlinear modeling	Uses handcrafted features only	Combines deep thermal enhancement with quantitative prediction
SVM	Effective on small datasets	Sensitive to parameter tuning and kernel selection	Directly models thermal biomarkers with interpretable outputs
XGBoost	Strong predictive performance	Reduced clinical interpretability	Generates continuous inflammation severity estimates linked to clinical scales
End-to-End Deep Regression	Fully automated prediction	Difficult to explain clinical decisions	Provides transparent relationship between thermal features and inflammation severity

Proposed DnCNN–MLR	Thermal enhancement + interpretable prediction	Requires feature extraction stage	Integrates denoising, feature enhancement, inflammation prediction, Joint Temperature Indexing, and MCDAI-based clinical decision support

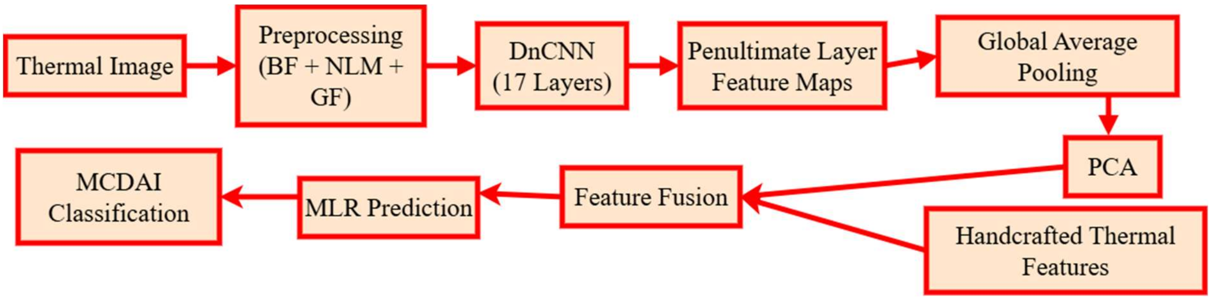

Figure S1. DnCNN layer architecture

Table S3. Five-fold cross-validation results for the knee joint thermal images

Fold	R^2	RMSE	MAE	Accuracy (%)
Fold 1	0.9987	0.0121	0.0098	99.2
Fold 2	0.9991	0.0114	0.0092	99.5
Fold 3	0.9990	0.0116	0.0094	99.4
Fold 4	0.9989	0.0118	0.0096	99.3
Fold 5	0.9993	0.0109	0.0088	99.6
Average	0.9990 ± 0.0002	0.0116 ± 0.0005	0.0094 ± 0.0004	99.4 ± 0.15

Table S4. Independent testing results for the knee joint dataset

Statistical Measure	Value
Number of Test Images	20
R^2 Score	0.9990
RMSE	0.0112
MAE	0.0091
MSE	0.00013
Pearson Correlation Coefficient (r)	0.9995
Accuracy (%)	99.5

Sensitivity (%)	99.3
Specificity (%)	99.6
F1-Score (%)	99.4

The paired t-test indicates statistically significant differences between all filter pairs ($p < 0.05$). The proposed hybrid filtering approach achieved significantly higher image quality than individual filters.

Table S5. Paired t-test analysis between preprocessing filters

Comparison	Mean Difference (dB)	t-value	p-value
Bilateral vs NLM	1.73	5.82	0.0021
Bilateral vs Guided	0.67	2.74	0.0285
NLM vs Guided	1.06	4.13	0.0068
Guided vs Proposed	3.84	11.42	<0.0001
NLM vs Proposed	2.78	8.96	<0.0001
Bilateral vs Proposed	4.51	13.87	<0.0001

Table S6. Five-fold cross-validation analysis of the PARA diagnostic framework

Condition	Fold	Accuracy (%)	Sensitivity (%)	Specificity (%)	MCDA Error
Normal	Fold 1	93.1	92.0	93.8	0.29
	Fold 2	94.2	93.3	94.7	0.32
	Fold 3	94.5	93.8	95.5	0.33
	Fold 4	93.7	93.0	94.9	0.30
	Fold 5	93.7	92.7	94.7	0.31
	Mean ± SD	93.84 ± 0.71	92.96 ± 0.78	94.72 ± 0.65	0.31 ± 0.02
Before Pregnancy	Fold 1	91.8	90.7	92.5	0.32
	Fold 2	92.6	91.4	93.2	0.36
	Fold 3	93.7	92.8	94.3	0.38
	Fold 4	92.8	92.1	93.9	0.34
	Fold 5	92.5	90.9	93.6	0.35
	Mean ± SD	92.67 ± 0.76	91.58 ± 0.83	93.49 ± 0.71	0.35 ± 0.03
During Pregnancy	Fold 1	89.3	88.1	90.2	0.40

	Fold 2	90.5	89.2	91.0	0.45
	Fold 3	91.6	90.5	92.2	0.49
	Fold 4	90.9	90.0	91.8	0.43
	Fold 5	89.8	87.9	91.6	0.43
	Mean ± SD	90.42 ± 0.88	89.14 ± 0.92	91.36 ± 0.81	0.44 ± 0.04
After Pregnancy	Fold 1	91.2	90.2	92.1	0.34
	Fold 2	92.5	91.4	92.9	0.38
	Fold 3	93.1	92.1	93.9	0.40
	Fold 4	92.7	91.8	93.4	0.36
	Fold 5	91.2	89.9	92.9	0.37
	Mean ± SD	92.13 ± 0.79	91.07 ± 0.86	93.04 ± 0.72	0.37 ± 0.03

Table S7. Methods adopted for obtaining noise-free or high-quality reference thermal images using a thermal camera

Method	Procedure	How Noise-Free Reference is Obtained	Suitable for Study
Multi-Frame Averaging	Capture multiple thermal frames (e.g., 20–100) of the same scene under identical conditions and compute the average image	Random noise varies between frames and is reduced through averaging	Simple, effective, widely used in thermal imaging research
Temporal Filtering	Acquire a sequence of thermal images and apply temporal smoothing or moving-average filtering	Reduces frame-to-frame fluctuations caused by sensor noise	Suitable for video-based thermal monitoring
Thermal Camera Calibration	Perform calibration using a blackbody source with known temperature	Corrects sensor drift, non-uniformity, and measurement errors	Improves temperature accuracy and image consistency
Non-Uniformity Correction (NUC)	Use built-in camera correction procedures to compensate for detector variations	Removes fixed pattern noise (FPN) across sensor elements	Standard feature in many thermal cameras
Blackbody Reference Imaging	Capture thermal images of calibrated blackbody targets	Provides a stable temperature reference for comparison	Generates highly reliable reference images

Thermal Phantom Imaging	Use thermal phantoms that simulate biological tissues or controlled heat patterns	Creates reproducible thermal distributions	Common in medical thermography validation
Controlled Environmental Acquisition	Maintain constant room temperature, humidity, distance, and background conditions during imaging	Minimizes external thermal disturbances	Easy to implement and improves image quality
Image Stacking	Combine multiple registered thermal images into a single composite image	Enhances signal-to-noise ratio (SNR)	Effective for static objects
Advanced Denoising Algorithms	Apply denoising methods such as Bilateral Filter, NLM, Wavelet Denoising, BM3D, or DnCNN	Estimates and suppresses noise while preserving thermal structures	Produces visually clean images
Synthetic Ground-Truth Generation	Use high-quality thermal images and artificially add known noise levels	Provides clean-noisy image pairs for algorithm evaluation	Useful for machine learning and denoising research
Radiometric Thermal Cameras	Use cameras with calibrated radiometric measurements and high thermal sensitivity	Reduces measurement uncertainty and improves image fidelity	Provides accurate temperature information
Hybrid Approach (Recommended)	Combine calibration, controlled acquisition, multi-frame averaging, and denoising	Simultaneously minimizes multiple noise sources	Produces the highest-quality reference thermal images

Table S8. Quantitative relationship between DnCNN features, MLR prediction, and MCDAI classification

Patient ID	Mean Temp (°C)	Max Temp (°C)	Temp Gradient	Entropy	DnCNN Feature Score	MLR Predicted Severity	Normalized Inflammation Scale (0–9)	MCDAI Category	Clinical Interpretation
P01	32.4	33.1	0.42	5.82	0.28	0.31	2.1	Low	Minimal inflammation
P02	32.8	33.8	0.57	6.01	0.35	0.38	2.8	Low	Mild inflammation

P03	33.4	34.6	0.76	6.24	0.49	0.52	4.2	Moderate	Localized inflammatory activity
P04	33.7	35.0	0.89	6.41	0.58	0.61	5.1	Moderate	Moderate disease activity
P05	34.1	35.8	1.12	6.63	0.71	0.74	6.0	Moderate	Active inflammation
P06	34.5	36.2	1.35	6.84	0.82	0.86	7.2	High	Significant inflammatory activity
P07	34.8	36.8	1.51	6.95	0.89	0.92	7.8	High	Severe inflammation
P08	35.2	37.3	1.72	7.08	0.96	0.98	8.5	High	Extensive inflammatory involvement
P09	35.5	37.8	1.91	7.16	1.03	1.05	8.8	High	Severe disease activity
P10	35.9	38.1	2.05	7.23	1.11	1.13	9.0	High	Very high inflammatory activity

- Mean Temperature (T_{mean})
- Maximum Temperature (T_{max})
- Temperature Gradient (G)
- Entropy (E)
- DnCNN Feature Score (F_{DnCNN})

then the general MLR model can be written as:

$$Y = \beta_0 + \beta_1 T_{mean} + \beta_2 T_{max} + \beta_3 G + \beta_4 E + \beta_5 F_{DnCNN} \quad (S1)$$

$$Y = -12.84 + 0.52 T_{mean} + 0.68 T_{max} + 1.95 G + 0.74 E + 2.31 F_{DnCNN} \quad (S2)$$

where Y is the raw inflammation severity predicted by the MLR model.

Table S9. Comparison of the proposed PARA framework with the recent literature

Ref.	Method	Data Type	Objective	Accuracy (%)	Limitations	Advantage of Proposed PARA Framework
[5]	CNN-Based RA Detection	X-ray Images	Joint abnormality detection	94.2	Radiation exposure, structural assessment only	Non-invasive thermal imaging and inflammation assessment
[6]	AI-Based Early RA Detection	X-ray Images	Early RA diagnosis	95.1	Limited physiological monitoring	Pregnancy-aware monitoring across disease stages
[9]	CNN Architecture	Clinical Imaging	Automated RA recognition	96.0	Black-box interpretation	Interpretable MLR-based severity prediction
[10]	CNN-LSTM	miRNA Dataset	Molecular-level diagnosis	97.3	Requires laboratory testing	No blood sample or laboratory biomarkers required
[11]	Deep Learning Framework	Clinical Dataset	RA diagnosis	96.8	Limited explainability	Combines deep features and thermal biomarkers
[13]	Optimized Deep Learning	Clinical Data	Therapeutic support	97.5	High computational complexity	Computationally efficient PCA–MLR integration
Proposed PARA	BF + NLM + GF + DnCNN + PCA + MLR + MCDAI	Thermal Images	Pregnancy-aware inflammation assessment	93.84 ± 0.71	Requires further clinical validation	Non-invasive, pregnancy-safe, interpretable, and decision-support oriented

Pseudo-Code of the Proposed PARA Framework

Algorithm 1: Pregnancy-Aware Rheumatoid Arthritis (PARA) Framework

Input:
Thermal images of elbow, hand/wrist, and knee joints

Output:
Inflammation severity score and MCDAI category

Step 1: Acquire thermal images under controlled conditions.

Step 2: Preprocess images
Apply Bilateral Filter (BF)
Apply Non-Local Means Filter (NLM)
Apply Guided Filter (GF)

Step 3: Select Guided Filter output as enhanced thermal image.

Step 4: Apply DnCNN
Remove residual thermal sensor noise
Generate denoised thermal image

Step 5: Extract deep features
Obtain feature maps from penultimate DnCNN layer
Convert feature maps into feature vectors

Step 6: Apply PCA
Reduce feature dimensionality
Retain discriminative principal components

Step 7: Extract handcrafted thermal features
Mean temperature
Maximum temperature
Temperature gradient
Standard deviation
Entropy
GLCM texture features

Step 8: Feature Fusion
Combine PCA features and handcrafted features

Step 9: Apply Multiple Linear Regression (MLR)
Predict inflammation severity score

Step 10: Normalize prediction to 0–9 scale

Step 11: MCDAI Classification
If score ∈ [0–3] → Low
If score ∈ [4–6] → Moderate
If score ∈ [7–9] → High

Step 12: Generate inflammation assessment report

Return:
Inflammation severity score
MCDAI category
Clinical decision-support recommendation

Table S10. Significance

Observation from Figure 9	Significance
Mean temperature increases with disease severity	Indicates increasing inflammatory activity
Maximum temperature rises progressively	Reflects localized joint inflammation
Temperature gradient becomes larger	Demonstrates stronger thermal asymmetry
Variance increases from normal to severe cases	Suggests heterogeneous heat distribution due to inflammation
Knee region shows the largest variation	Indicates high sensitivity for RA monitoring
Consistent trends across joints	Validates the use of thermal biomarkers for MLR prediction
Distinct separation among severity levels	Supports MCDAI-based inflammation classification

Table S11. Summary of the literature survey and research gap

Research Area	Key Findings	Research Gap
Traditional Therapies [1]	Effective in reducing inflammation and pain.	Limited support for continuous disease monitoring.
X-ray-Based Diagnosis [2,5,7,15]	Detects joint damage and structural abnormalities.	Radiation exposure and poor early inflammation detection.
AI/ML-Based Prediction [4,6,9,13]	Improves diagnostic accuracy and disease prediction.	Limited interpretability and pregnancy-specific assessment.
Genetic and Molecular Studies [8,10–12,16,17]	Identifies biomarkers and disease susceptibility.	Expensive and requires specialized laboratory facilities.
Thermal Imaging [14]	Non-invasive and radiation-free disease assessment.	Limited application for RA monitoring during pregnancy.
Clinical Disease Activity Assessment [18–20]	Biomarkers and disease activity indices remain important.	Dependence on laboratory testing and clinical evaluation.
Overall Inference	Existing methods improve RA diagnosis and monitoring.	Need for a pregnancy-aware, non-invasive, interpretable AI framework using thermal imaging for RA assessment.
